# Assessment of Gaze Fixations and Shifts in Children with Cerebral Palsy: A Comparison of Computer- and Object-Based Approaches

**DOI:** 10.3390/jcm14072326

**Published:** 2025-03-28

**Authors:** Tom Griffiths, Michael T. Clarke, John Swettenham

**Affiliations:** 1 School of Computing, University of Dundee, Dundee DD1 4HN, UK; 2 Department of Speech, Language and Hearing Sciences, San Francisco State University, San Francisco, CA 04132, USA; michaelclarke@sfsu.edu; 3 Division of Psychology and Language Sciences, University College London (UCL), London WC1E 6BT, UK; j.swettenham@ucl.ac.uk

**Keywords:** cerebral palsy, functional vision, eye tracking, fixation, strabismus

## Abstract

**Background/Objectives**: Gaze behaviours, such as fixation on single objects, and switching gaze between two objects are important for signaling messages, making choices or controlling a computer for children with cerebral palsy (CP) and similar movement disabilities. Observing these behaviours can be challenging for clinicians, with a lack of agreement on how they can be objectively quantified or rated. **Methods**: This study compares two methods of eliciting and observing gaze behaviours: a computer presentation using an eye tracker and an object presentation scored by two independent observers in order to explore the utility of each to clinicians working in this area. Children with CP (*n* = 39) attempted single-target fixation (STF) and target–target fixation shift (TTFS) tasks using both presentations and the results were compared. **Results**: Six children were unable to calibrate the eye tracker to the accuracy level required. Significantly higher scores for both STF (81.3% object presentation and 30.3% computer presentation, *p* < 0.01) and TTFS (70.1% and 26.9%, *p* < 0.01) were seen on the object presentation, with children’s performance not predicted by developmental age, severity of CP or presence or absence of strabismus. It is not possible to definitively state which method gives the “correct” result; however, the difference in reported success rate merits further discussion. **Conclusions**: Whilst eye tracking may present an “entry barrier” for some children in terms of its accuracy and calibration requirements, object presentation carries with it the risk of over-interpreting children as having fixated. Conversely, eye tracking may be better at recording fixations in children with strabismus, where object-based paradigms may offer more flexible administration for clinicians. The variability in children’s performance on both presentations underlines the risk of assuming these skills to be present and the importance of assessing gaze behaviours in individual children.

## 1. Background

Cerebral palsy (CP) is a neurodevelopmental condition primarily characterised by limitations of movement and difficulties maintaining posture, beginning in early childhood and persisting throughout a person’s life [1]. For children with CP whose motor impairments are more severe, there is a greater incidence and severity of oro-motor difficulties that may in turn limit or preclude the use of clear speech [2]. Communication difficulties of this nature are known to negatively impact children’s participation in community and family life [3] and can impact learning, curriculum access and school inclusion [4]. Children with severe motor impairments who cannot rely on speech to communicate and whose motor disabilities preclude the use of volitional pointing using upper or lower limbs may make use of controlled eye movements as a means of signalling messages—a method often termed eye pointing [5]. Eye pointing as a communication modality falls under the group of strategies, techniques and devices commonly referred to as augmentative and alternative communication (AAC), which are used to support children’s expressive communication and language learning. Eye pointing may be a method to access and use formal communication systems, which are explicitly introduced and taught, such as the use of real-world objects, paper-based printed material such as communication books, boards and photographs, and powered voice output communication aids based on mainstream or bespoke computer systems [6,7]. In particular, those involved in the selection of AAC systems may consider reliable eye pointing to be a cue to investigate the use of eye-gaze technology for communication and computer control, a method of controlling a computer using the movement and rest of gaze to select onscreen items [8,9]. However, not all children with CP appear able to make full use of their gaze for reliable eye pointing or eye-gaze control, and it has been observed that the component skills needed to make use of either are not routinely assessed and described, with significant implications for children, families and clinicians. The lack of consistent understanding of the skills needed to use gaze for either of these purposes presents the risk that it will be assumed that children can make use of these skills, creating unrealistic expectations around children’s communication and/or goals for intervention that do not meet children’s real needs [10,11].

### 1.1. Gaze Fixation

In both eye pointing and eye-gaze access, one key component is children’s making use of purposeful and sustained fixations as a substitute for a “point”, where the sustained fixation of the gaze on an object may substitute indicating choice or directing attention. When applied to eye-gaze control, gaze acts as a method of selection: an input device that allows direct manipulation of a cursor and selection of objects in a similar way to using a mouse or touchscreen. Both eye pointing and eye-gaze control require children to make use of purposeful gaze fixations and gaze shifts to engage with their environment, or to respond to stimuli.

The reading or interpretation of fixations and shifts, either by a communication partner or an eye tracker, is considerably more difficult than the interpretation of a finger point. The high rate of impairment of diverse aspects of the visual system seen in children with CP [12,13] may be one barrier to easily identifying where a child is fixating. Strabismus or “squint”, a condition where the visual axes of both eyes are not parallel and the orientation of one eye deviates from the point of visual fixation, is one issue that may complicate or limit an observer’s ability to tell where a child is looking. Strabismus occurs in up to 5% of the population, but a recent systematic review puts the prevalence of strabismus in children and young people with CP at 48% [14]. The presence of strabismus has been shown to be related to poorer quality fixation data in eye-tracking studies [15,16]. In behavioural studies, human observers may experience difficulties in accurately reporting the target of gaze fixations in children with strabismus [17].

Other challenges in reading or interpreting fixations and gaze shifts as deliberately communicative arise from the difficulty in reliably distinguishing instances of gaze with communicative intent from the use of gaze to simply “look at” or examine something. The use of gaze in developmentally younger children may be subject to conscious or unconscious over-interpretation by observers, with ‘looking at’ often described as ‘pointing to’ without full consideration of whether the gaze is intended as a communicative signal [5]. Conscious interpretation of a child’s looking as a purposeful act is an established therapeutic approach when working with children at the early stages of communication development [18], albeit one that requires a recognition on the part of the observer that these behaviours are being “scaffolded” or shaped into a communicative act [19]. Sargent and colleagues [5,20] propose that an observer’s confidence in interpreting gaze as communicatively purposeful can be increased by the child looking between the object of interest and their communication partner in a triadic gaze shift (object–partner–object, or partner–object–partner). Reading gaze shifts as communicatively purposeful, therefore, requires a level of interpretation that can complicate the task of observing and recording them.

The challenge of reliably interpreting gaze as communicatively purposeful is analogous to the “Midas Touch” problem in human–computer interaction [21,22,23], which proposes that systems are prone to over-selectivity when they use the same channel to both receive information and transmit signals. In the classic presentation of this problem in gaze interaction, a user may make selections whilst simply looking at an item, whether they intended to or not. Similar issues are at play with the use of gaze for communication: a child may be inspecting an object, or preferentially attending to a more visually salient object; however, this may be interpreted as an episode of directed gaze and construed as a selection or choice by an observer.

### 1.2. Observation and Recording of Fixations

The use of fixation can be variable in the population of children with CP [5,24,25]; however, it has been observed that children’s ability to use gaze fixations and gaze shifts is not routinely or systematically documented by clinicians or professionals involved in communication or computer access assessment [9,20]. The term “functional vision” is often employed to describe how a person functions in vision-related activities, as distinct from “visual function”, which describes the structure and functioning of the eyes and the visual pathways. Functional vision skills are those related to the use of “vision to perform critical or meaningful tasks” [26], and how well a child with CP can make functional use of their vision may be important in their development of communication and control. However, comparatively little attention is paid to observing or recording difficulties with functional vision skills compared with reporting visual functions and impairments, such as acuity reduction or strabismus [13,24,27]. One possible reason for this, as proposed by Deramore Denver and colleagues, is that there is no standardised instrument to measure them and no quantitative count or measure that can be applied to these skills, in contrast to a visual impairment, such as acuity reduction [13]. Another reason these skills are not routinely observed and recorded may be the perception that all aspects of vision are the prevail of vision professionals such as ophthalmologists, and hence clinicians and educators working with AAC may consider descriptions of vision “off limits” [20,28]. Work carried out by Clarke and colleagues highlights that, without undermining the important work of vision professionals, there is scope for non-vision specialists to make potentially informative observations of children’s functional use of vision for communication in the form of gaze fixations and gaze shifts [29,30]. This group consulted a variety of different stakeholders involved in the assessment of communication (including representation from parents and support workers of children with CP, alongside clinical representatives from the fields of clinical paediatrics, psychology, allied health professions, clinical science, orthoptics and optometry) on the best methods to observe various functional vision skills, including eye pointing. Whilst the group acknowledged that eye pointing includes coordination of a child’s visual, social, motor and cognitive abilities, it was felt pragmatic to focus on describing “observable looking behaviours” [30], including those that represent a progression of skills, which build towards eye pointing, such as single-target fixation and target–target fixation shift. Similarly, Sargent and colleagues [20] demonstrated that parents can provide useful descriptions of their child’s patterns of fixation when asked questions as part of a structured clinical history taking.

In this paper, we focus on children’s basic ability to fixate and shift gaze—more specifically, the fixation of gaze on a single target (single-target fixation) and the switching of fixation between two targets (target–target fixation shift). This paper seeks to explore and discuss two potential methods of observing and recording directed gaze in children with CP: a computer presentation of onscreen stimuli, combined with an eye-tracking camera to record gaze position data, and the presentation of objects by an observer.

### 1.3. Computer Presentation

Computer displays are a flexible way to present stimuli with a range of controllable attributes (colour, size, position, complexity, etc.). Combining a computer display with an eye tracker offers the possibility of recording the eye movements of the user in response to what is presented on screen. Because of this, eye trackers have seen widespread adoption in interaction design, usability evaluation and marketing, as well as in disciplines such as neuropsychology, cognition research and vision sciences [31]. The use of eye-tracking technology in research may be a useful tool to provide objective and reliable measures of typically developing children’s looking behaviours [32] and to tailor assessments of vision to individuals [33]. Studies of children’s fixation patterns to provide insight into the development of social orienting [34,35] and false beliefs [36,37] are found in the literature on autistic children [38]. The technology is considered well-suited to the task of assessing these children, especially those children who are developmentally younger, since it can be combined with assessments that do not require understanding of language and that use children’s visual orienting or fixation to assess their performance, whilst placing minimal demands on the child [39,40]. The technology is less frequently used in the assessment and monitoring of children with CP, although it shows the potential to provide greater insight into the implicit skills of this population, for example, through tracking anticipatory gaze behaviours [37].

Computer presentation of stimuli has been identified as one potential method to observe and record children’s fixations and gaze shifts, and professionals working with children with severe movement limitations report an interest in being able to record and analyse these [41]. An eye tracker potentially provides an objective method to determine when fixations and gaze shifts have occurred. Although the technologies and physical hardware are often similar, eye-tracking technology, for the purposes of this paper, is distinguished from eye-gaze technology (sometimes known as eye-control technology) in that it is a passive tracking technology for collecting data, as opposed to an active control method. Given this use case, eye tracking tends to have both a higher spatial and temporal resolution, enabling the capture and detailed analysis of eye movements, including subtle eye movements that might otherwise be difficult to observe [31]. Eye-gaze technology is used to control a computer, typically by emulating mouse movement, and, therefore, has lower requirements for fine-grained accuracy or measurement, focusing instead on providing instantaneous feedback to the user on gaze position and onscreen item selection at minimal processing cost.

#### Calibration

**Figure 1:**
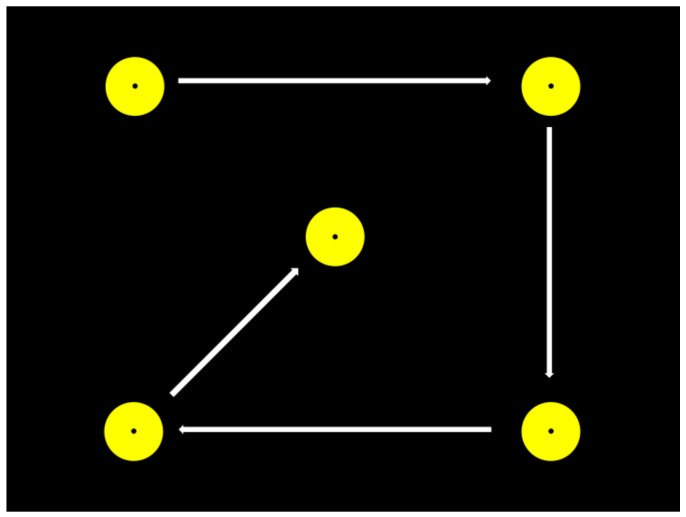
Graphical representation of a five-point calibration procedure with arrows showing the direction in which the stimulus moves, stopping at each location to allow the eye tracker to sample the position of the users’ eyes.

In order to produce accurate data on a user’s eye movements, a calibration procedure must be carried out prior to use to ensure that the gaze point data collected are a valid representation of where the user’s line of sight intersects with the device’s display [42,43]. This is necessary because every user’s eyes are subtly different in shape and size, meaning that the models and algorithms available to the eye tracker are not universally accurate and need adjusting for each user. Most calibration procedures follow a similar paradigm: visual targets are displayed at known locations on the display (Figure 1), with the system recording the location of the user’s gaze point relative to these targets [44]. Information on the accuracy and precision of recorded gaze points can then be factored into the tracking algorithm to improve accuracy. The rate of successful calibration is known to vary in both children and adults: children with no reported disabilities have been shown to calibrate successfully 92–100% of the time [39], whereas children with intellectual disabilities have reported calibration rates of 83% [45]. The authors are not aware of similar figures for children with CP reported in the published literature.

The conventional calibration paradigm is, in essence, a fixation task. Nyström and colleagues [46] note that calibration requires fixation on a single point in various locations and that this may present an issue for some user groups, including children and people with certain disabilities, who have difficulties maintaining attention or fixating on a single item may be difficult to calibrate owing to the device’s needing to pass a pre-specified data threshold to confirm a successful calibration.

### 1.4. Object Presentation

Presenting objects as stimuli is an established part of research and clinical assessment. Many developmental, psychological, vision or language assessments rely on the use of objects as stimuli to elicit specific responses. Observation of a child’s eye movements and their orientation to light sources and other visually salient stimuli are common in early developmental assessment, and children’s difficulties with these activities may be a cue for clinicians that further investigation of a child’s vision or neurology may be required [47]. Of relevance to the current paper, measurement of visual acuity using materials such as the Keeler Infant Acuity Cards (Keeler Ltd., Windsor, UK) or the Cardiff Acuity Test (Kay Pictures Ltd., Tring, UK) use cards that display black and white stripes or picture outlines. These materials measure resolution acuity and depend upon the child’s preference to look at a high-contrast target rather than a grey background—sometimes referred to as a “preferential looking test” [48,49]. Tests, such as the Near Detection Scale [50,51], make use of objects as stimuli to explore early visual-orienting and visual-search skills in babies and infants. The use of objects to observe children’s use of vision in functional tasks has also been proposed by several researchers [29,30,52].

The presentation of objects has been identified as another method to support the observation of children’s directed gaze. The use of hand-held, real objects is familiar to clinicians and it may be easier for observers to vary and control the presentation of hand-held stimuli. Their use does not require any particular prior knowledge or familiarity with technology on the part of the observer.

### 1.5. Aims and Research Questions

This paper presents the results of trials using both computer presentation and object presentation of stimuli to observe children’s directed gaze, in particular, single-target fixations and target–target fixation shifts. Inevitably, the two presentation methods are not identical and they are not perfectly matched; however the aim is to compare both from the standpoint of clinicians needing to observe these behaviours in children with CP. The following research questions are addressed:

- What advantages do computer and object presentations of fixation and gaze-switching tasks offer clinicians seeking to observe these skills in children with CP?
- Are children with CP able to calibrate an eye tracker with enough precision to allow the gathering of reliable data on their eye movements?
- How well does data from an eye tracker match data from human observations?

## 2. Materials and Methods

### 2.1. Participants

Children were recruited for the study from special schools in London and a specialist communication clinic based at a London hospital. Parents were given an information leaflet and invited to make contact with the research team if their child met the following inclusion criteria:

- 4-limb (bilateral) CP requiring wheelchair use (GMFCS categories IV and V);
- chronological age 40–160 months;
- language understanding/intellectual ability assessed or reported to be within the 12–54 month range;
- hearing levels adequate for speech recognition.

The age range for language and intellectual ability was dictated by the age range of the two assessment tools used in the background screening of participants. Children were ineligible for participation in the study if any of the following were noted:

- severe or profound sensorineural hearing loss;
- visual acuity loss not due to correctable refractive errors and sufficient to preclude object resolution at 30 cm distance;
- confirmed oculomotor dyspraxia;
- untreated or uncontrolled epilepsy.

A total of 66 children with CP (34 male, 32 female) chronologically aged between 3 years 4 months and 12 years 8 months (*M* = 91.1 months, *SD* = 28.59 months) were enrolled in the study. All children completed background screening tasks to verify that they met the inclusion criteria and to provide demographic data. Functional motor abilities were classified using the *Gross Motor Function Classification System—Expanded and Revised* (GMFCS; [53]). Receptive language was assessed using an adapted version of the *Pre-School Language Scales UK—4th Edition* (PLS-4UK; [54]). Children’s non-verbal cognition was assessed using the visual reception scale from the *Mullen Scales of Early Learning* (MSEL; [55]), which assesses cognitive performance independent of language skills [56]. All assessments were carried out by clinicians with extensive experience assessing children with CP. During the PLS4-UK and MSEL assessments, it was noted whether the children’s vision was not at a level sufficient to engage with the test materials. Following background measures, the following children were excluded from the study:

- language and/or cognition fell below the level for inclusion (*n* = 12);
- language and/or cognition fell above the level for inclusion (*n* = 3);
- GMFCS Level III or below (*n* = 2);
- chose not to participate or too unwell to participate (*n* = 2);
- visual impairment considered too severe to detect test materials (*n* = 8).

Therefore, 39 children took part in the observations of directed gaze. The characteristics of these children are summarised in Table 1.

There was a large mean difference between children’s chronological age and the age equivalents reported on the PLS4-UK and MSEL (Table 2). Age-equivalent scores on these assessments were significantly correlated (*r*(37) = 0.925, *p* < 0.001). Language age equivalent, as reported on the PLS-4UK, was used for analysis.

Children were seen either at home, in school or in a dedicated behavioural laboratory space. Separate areas for administration of the computer-presented and object-based tasks were set up. Every effort was made to minimise distractions in the environment. Each child was assessed for approximately 90 min with regular breaks, ideally within one session although multiple sessions were permitted as required. Children recruited to the study attended sessions in their most supportive seating system. If a child became fatigued, distressed or indicated that they would like to stop, the session was terminated.

### 2.2. Setup and Procedures

All materials used in this study were reviewed to ensure that they would be visible to participants meeting the inclusion criteria. The services of a specialist Paediatric Optometrist were recruited to verify that the materials used were accessible to children with low levels of vision, including those described as having only detection vision:,characterised by the ability to detect a single item against a plain backdrop without the need to resolve adjacent visual targets [57]. The use of materials requiring only detection vision allowed for the inclusion of the largest possible number of children, which in turn allowed the researchers to make observations and gather data on children considered to be representative of the clinical populations being considered for trials of eye-gaze control technology.

Both the computer and object presentations comprised a single-target fixation (STF) task and a target–target fixation shift (TTFS) task. The computer presentation was preceded by a calibration procedure.

#### 2.2.1. Computer Presentation Setup

A *Tobii T60* display-mounted eye tracker, connected to a laptop running the *Tobii Studio* software (v 3.4.5), was used for the computer-presented tasks. All stimuli were presented on the *T60′*s 17″ display (1280 × 1024 px resolution, 4:3 aspect ratio) and eye-tracking data were sampled at 60 Hz. The children’s positions relative to the eye tracker were controlled by positioning the device at a standard distance of 60 cm, with the eye-tracking camera at a 45° angle relative to the child’s horizontal eye-line. Positioning was checked using the software’s built-in positioning guide. Stimuli displayed on the device were of a constant size for all trials, with an absolute measurement of 3.5 cm × 3.5 cm on the display, giving a visual angle of 3.3° when viewed at a distance of 60 cm.

#### 2.2.2. Calibration

All children attempted a five-point calibration of the eye tracker following the manufacturer’s guidelines and using the software’s default settings of a yellow circular target moving sequentially between the four corners and the centre of the screen. The target advanced automatically through each of the five positions once the device had collected enough samples of the child’s gaze point relative to the known coordinates of the target. Calibration was deemed unsuccessful if the device was unable to reliably cluster the sampled gaze points, if samples were too far from the target centre or if not enough samples could be acquired during the set time period. If calibration was unsuccessful after three attempts, then a two-point “Infant Calibration” was attempted: an animated duck with an accompanying bell sound, providing increased visual and auditory salience. This stimulus was controlled by the experimenter, allowing more flexibility to ensure the child was focused on the screen before triggering the software to capture the gaze points. This calibration method is suggested by the manufacturer for those who may have difficulties following instructions given by an assessor during calibration [58]. Where children presented with difficulties retaining their head at the midline, parents were asked to gently support the child’s head during the calibration process. It is known that the accuracy of an eye tracker is greater when test conditions are as similar as possible to the conditions occurring during calibration [46], so the tasks took place immediately after the calibration procedure had been completed. Where calibration was unsuccessful, children did not proceed to computer presentation tasks, since uncalibrated data are considered invalid. Data on whether children were able to calibrate and which calibration protocol was used, and any observations made about a child’s behaviour or performance during the task were recorded in field notes kept by the researcher.

#### 2.2.3. Computer Presentation Tasks

**Figure 2:**
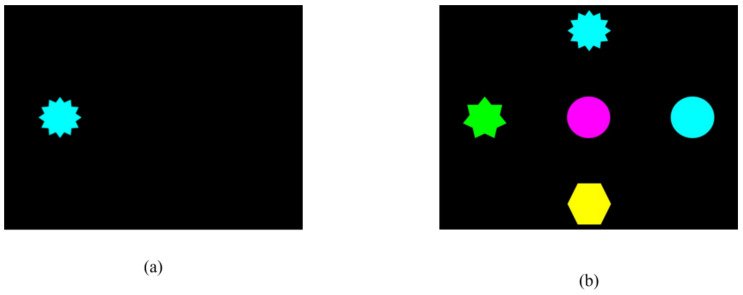
The eye tracker version of the STF task showing (**a**) an example of a trial and (**b**) the five possible positions and shapes of the stimuli.

The STF task began with a blank screen. Children’s attention was drawn to the centre of the screen by pointing or tapping a finger in the centre, alongside verbal instruction (e.g., “Let’s look for some shapes!”). No fixation cross or similar prompt stimuli was used for this experiment to avoid any effect caused by the need to disengage from one stimulus and switch to another. Using the *Tobii Studio Live Viewer*, it was ensured that the child’s gaze point was within the screen area before the stimulus appeared in one of five locations (Figure 2). The stimulus was present onscreen for six seconds. The location where the stimulus appeared was randomised and counterbalanced. Ten trials were presented to each child. For each trial, the software recorded whether or not the child had fixated on the stimulus. Children scored “1” if a fixation was recorded and “0” if no fixation was recorded (Maximum score = 10).

**Figure 3:**
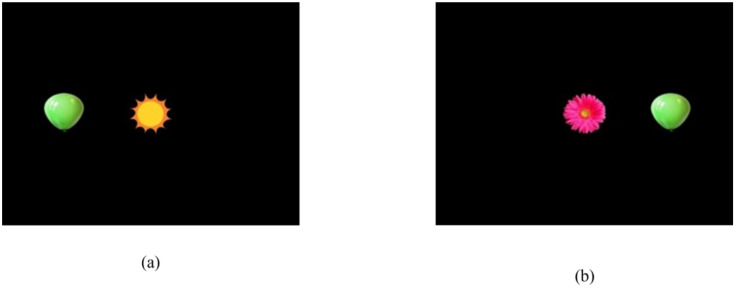
The eye tracker version of the gaze-switching task showing the two central stimuli (sun and flower) and the two positions of the peripheral stimuli (**a**) left and (**b**) right.

Following a short break, children were presented with the TTFS task, beginning with a blank screen. Once the researcher was sure from checking the *Live Viewer* that the child’s gaze was oriented towards the screen, a central stimulus appeared—either a yellow and orange sun or a pink flower (Figure 3). Both were sized to match the stimuli in the STF task. However, in this experiment, the stimulus grew and shrank around a central point, giving the visual impression of “pulsing” in and out. Once the *Live Viewer* showed that the child’s gaze point was located in the region of the central stimulus, the peripheral stimulus was triggered by the researcher and presented for a period of six seconds, after which the display went blank until the researcher triggered the next trial. The peripheral stimulus appeared either to the left or right, offset by approximately 7° of visual angle when viewed at a distance of 60 cm, and was always a green balloon, sized to match the central stimulus (Figure 3).

The location (left or right) where the peripheral stimulus appeared was randomised and counterbalanced. Four trials of this procedure were conducted. For each trial, the software recorded whether or not the child had fixated on the peripheral stimulus. Children scored “1” when the device recorded a gaze shift resulting in a fixation on the peripheral stimulus and scored “0” if no gaze shift was recorded within six seconds of the onset of the peripheral stimulus (maximum score = 4).

#### 2.2.4. Object Presentation Setup

**Figure 4:**
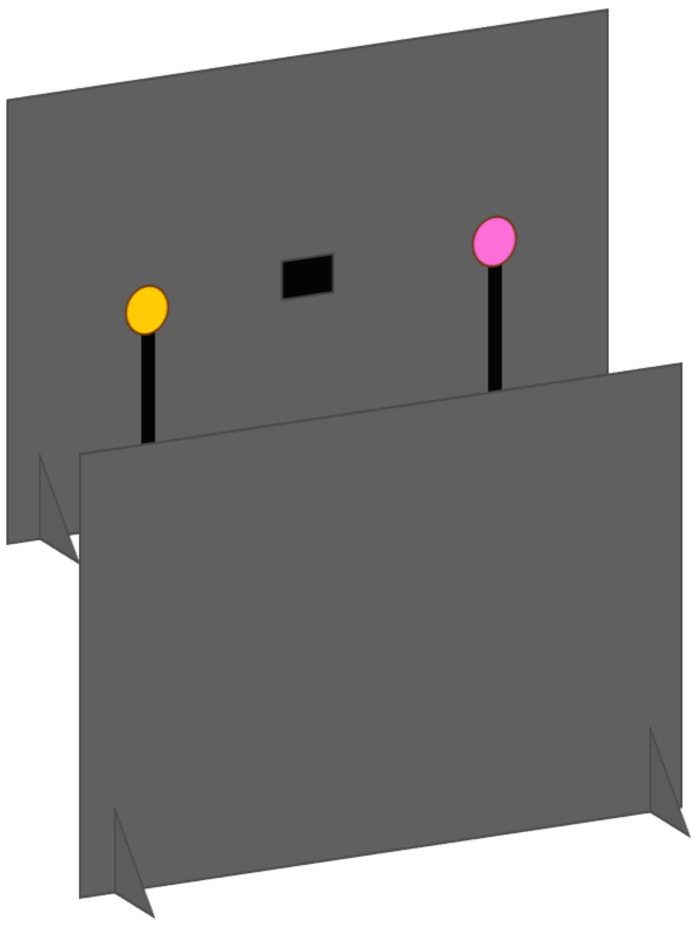
The object-based presentation setup. Chairs holding the upper board in place are not shown to reduce image clutter.

**Figure 5:**
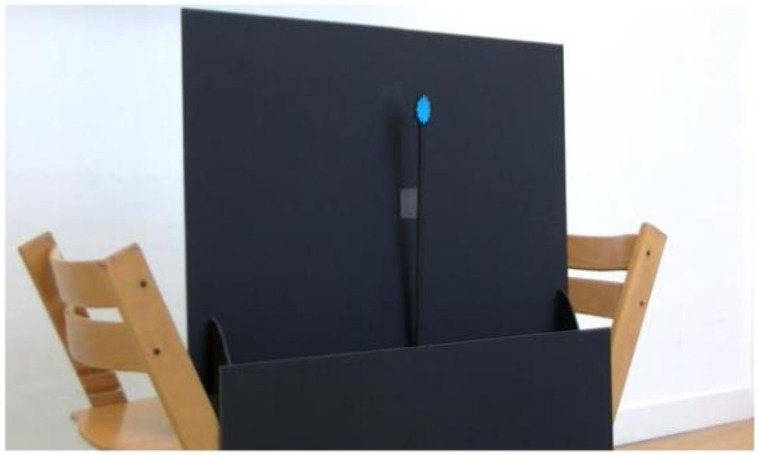
The object-based setup with an STF stimulus displayed—the researcher is fully concealed behind the boards.

A bespoke setup was developed by the researchers and clinical colleagues for the object-based tasks (Figure 4 and Figure 5). Following the design principle that all materials should be easily replicable for clinicians, the setup used two black foam-board sheets of A1 size (84.1 × 59.4 cm) positioned in landscape orientation, one above the other at a slight offset to create a channel of roughly 15 cm, allowing the stimuli to be presented at the child’s eye level by a researcher kneeling behind the setup. Boards were kept upright by “feet” made from the same foam board. The lower board stood on the floor, with the upper board positioned on two chairs. The upper board had a hole cut in the centre, which was shielded by a layer of black mesh, allowing the researcher to view the child’s eye movements whilst remaining obscured and not providing gaze cues or distracting the child. The stimuli used were brightly-coloured shapes, a sun, a flower and a balloon, all of around 5 cm diameter, mounted on sticks approximately 40 cm in length, cut from the same foam-board material (Figure 4).

Object presentation tasks were scored by two observers, each blinded to the other’s scoring, to allow the reliability of observations to be checked. Both observers were clinicians with clinical experience in observing and documenting the use of vision as a communication or signalling modality in children with CP. The first observer was positioned behind the boards so that they could see through the hole and observe the child’s gaze behaviours from the best possible vantage point—at eye level, directly opposite the child. This observer controlled the presentation of the stimuli. The second observer stood behind the board setup and slightly to one side so as not to distract the child.

#### 2.2.5. Object Presentation Tasks

The STF task began with the child’s attention being drawn to the boards by a member of the research team. Once the child was observed to be looking towards the boards, a single stimulus was raised, with the coloured side facing away from the child and therefore hidden, into one of five positions (left, right, top, bottom, centre). The stimulus was then rotated so that the coloured side became visible and was displayed for 6 s (Figure 5) before being turned back. The stimulus was then presented in the next location. The stimulus was presented twice in each location for a total of ten trials, with the order of presentation randomised and counterbalanced, ensuring that it did not appear at the same location twice in a row. Scorers were asked to indicate whether a fixation of two seconds or longer had been achieved. Children were, therefore, given a score of “1” if they fixated on the stimuli within six seconds and a score of “0” if a fixation did not take place within this time (maximum score = 10).

The TTFS task also began with the child’s attention being drawn to the boards. A stimulus was raised into the centre of the upper board, just below the observation window in order not to obscure the first observer’s view. Once the first observer was confident that the child was directing their gaze to this stimulus, a peripheral stimulus was displayed for six seconds, either to the left or the right. The central stimulus remained in place. The peripheral stimulus was presented twice at each location, for a total of four trials. Children were given a score of “1” if they fixated on the peripheral stimulus within six seconds and a score of “0” if a fixation did not take place within this time (maximum score = 4).

### 2.3. Analysis

The *Tobii Studio* software was used to calculate the location of the gaze point relative to defined areas of interest (AOI) and the duration of fixations on each trial of the computer presentations. For each trial, an AOI was overlaid on the area of the stimulus. The software then analysed whether any instance of fixation had taken place within the AOI in the six seconds following the onset of the stimulus. A default fixation—defined as gaze data being sampled within a 30 px area for 100 ms—was used for all analyses. Fixations with a reaction time to fixation (RTF) of less than 0.1 s were removed from the analysis as such a short RTF was likely to indicate that the child’s gaze point was already within, or very near, the AOI, at the onset of the stimulus.

The object presentation tasks were scored live by the two observers during the sessions.

The data from the two observers were checked for inter-rater reliability. All data were transferred to IBM SPSS (v.29) software for statistical analysis. We used *t*-tests for between-group comparisons. The relationship between variables (e.g., language age equivalent scores and percentage of fixations) was analysed using Spearman’s rank–order correlation (Spearman’s rho, *r*^s^) given the small sample size. We used the chi-squared test (*χ*^2^) to evaluate the association between categorical variables (GMFCS and calibration) and Fisher’s Exact Test (FET) when the sample size was too small for chi-squared. We assessed the level of agreement between two independent observers using Cohen’s kappa (K). All *p*-values are stated for the relevant statistics, and unless otherwise stated, a value of <0.05 was considered to indicate a statistically significant result. Adjustments for multiple comparisons were not required.

## 3. Results

### 3.1. Eye Tracker Calibration

Six children (15.4%) were unable to calibrate using either the standard or infant calibration. Observed reasons for non-calibration included an inability to fixate on the target, non-attention to the device, difficulties with head control or maintaining posture, and non-compliance with the activity. There was no statistically significant difference between the language age equivalents of the children who calibrated (*n* = 33, *M* = 26.4 months, *SD* = 11.2) and those who did not (*n* = 6, *M* = 27.0 months, *SD* = 10.0) (*t*(37) = 0.11, *p* = 0.95, *d =* 0.05). There was no significant association between children’s GMFCS level and their calibration (*χ*^2^(1) = 1.42, *p* = 0.23).

### 3.2. Computer Presentation Tasks

We analysed the data for all 39 children on the computer-presented assessment. As calibration is part of the procedure for assessing children on the computer-presented task, the six children who could not calibrate were scored as having demonstrated no examples of STF or TTFS using the computer-presented assessment. They were, therefore, given a score of zero for both tasks.

#### 3.2.1. Single-Target Fixation (STF)

The mean STF percentage was 30.2% across 10 trials (*SD* = 30.3%, *Range* = 0–100%). There was no statistically significant relationship between language age equivalent and percentage of recorded STF (*r*^s^(37) = 0.03, *p* = 0.88). There was no significant difference in percentage of recorded STF between children with strabismus and children without strabismus (strabismus: *n* = 12, *M* = 27%, *SD* = 28.8%; no strabismus: *n* = 27, *M* = 31.6%, *SD* = 31.3%) (*t*(37) = 0.43, *p* = 0.67, *d = 0*.15). Thirteen children did not demonstrate any STF on the computer-presented trials (6 failed to calibrate, and 7 calibrated but recorded no fixations on the trials). These children did not differ from the rest of the group in language age equivalent (fixators: *M* = 25.6 months, *SD* = 10.4 months; non-fixators: *M* = 28.4 months, *SD* = 12.0 months) (*t*(37) = 0.75, *p* = 0.46, *d* = 0.25) and they were no more likely to present with strabismus than those who could fixate (*p* = 0.71, Fisher’s Exact Test, FET).

#### 3.2.2. Target–Target Fixation Shift (TTFS)

The mean TTFS percentage was 26.9% across 4 trials (*SD* = 36.5%, *Range* = 0–100%). There was no statistically significant relationship between language age equivalent and percentage of recorded TTFS (*r*^s^(31) = 0.07, *p* = 0.67). There was no significant difference in the percentage of recorded TTFS between children with strabismus and the children without strabismus (strabismus: *n* = 12, *M* = 22.9%, *SD* = 37.6%; no strabismus: *n* = 27, *M* = 28.7%, *SD* = 36.5%) (*t*(37) = 0.45, *p* = 0.65, *d* = 0.16). Twenty-one children did not demonstrate TTFS on the computer-presented trials (6 failed to calibrate, and 15 calibrated but recorded no fixation shifts on the trials). These children did not differ from the rest of the group in language age equivalent (shifters: *M* = 27.7 months, *SD* = 11.6 months; non-shifters: *M* = 25.6 months, *SD* = 10.4 months) (*t*(37) = 0.59, *p* = 0.56, *d* = 0.19) and they were no more likely to present with strabismus than those who could shift fixations (*p* = 0.74, Fisher’s Exact Test, FET).

A significant correlation was demonstrated between performance on the STF and TTFS tasks when presented on computer (*r^s^*(37) = 0.68, *p* < 0.001).

### 3.3. Object Presentation Tasks

All children (*n* = 39) attempted the object presentation tasks.

#### 3.3.1. Single-Target Fixation (STF)

The mean STF percentage was 81.3% across 10 trials (*SD* = 27.1%, *Range* = 0–100%). Two children demonstrated no STF on the object presentation tasks. There was a significant positive correlation between language age equivalent and percentage of observed STF (*r_s_*(37) = 0.34, *p* = 0.03). Children with strabismus had a lower mean percentage of STF than those without (strabismus: *n* = 12, *M* = 69.2%, *SD* = 37%; without strabismus: *n =* 27, *M* = 86.7%, *SD* = 19.8%) although this difference was not significant at the 0.05 level (*t*(37) = −1.57, *p* = 0.06, *d* = 0.59) the effect size was moderately high. There was substantial overall agreement between the two independent observers on whether STF was observed (*K* = 0.678).

#### 3.3.2. Target–Target Fixation Shift (TTFS)

The mean TTFS percentage was 70.1% across 4 trials (*SD* = 36.1%, Range = 0–100%). There was no significant correlation between language age equivalent and percentage observed TTFS *(r_s_*(37) = 0.255, *p* = 0.117). Children with strabismus had a lower mean percentage of observed TTFS compared to those without (strabismus: *n* = 12, *M* = 66.7%, *SD* = 28%; no strabismus: *n* = 27, *M* = 85.3%, *SD* = 23.1%). Although this difference was not significant at the 0.05 level (*t*(37) = 1.93, *p* = 0.06, *d* = 0.76), the effect size was moderately high. There was moderate overall agreement between the two observers on whether TTFS was observed (*K* = 0.590).

A significant correlation was demonstrated between performance on the STF and TTFS tasks in the object presentation condition (*r_s_*(37) = 0.339, *p* = 0.035).

### 3.4. Comparison of Computer and Object Presentations

Next, we compared the scores obtained from the same group of children on the two methods of observation. Children demonstrated a significantly higher successful STF percentage on the object-presented trials (*M* = 81.3%, *SD* = 27.1%) than on the computer-presented trials (*M* = 30.2%, *SD* = 30.3%) (*t*(38) = 7.8, *p* < 0.01, *d = 1.3*). They also demonstrated a significantly higher successful TTFS percentage on the object-presented trials (*M* = 70.1%, *SD* = 36.1%) than on the computer-presented trials (*M* = 26.9%, *SD* = 36.5%) (*t*(38) = 6.5, *p* < 0.01, *d* = 1.0). There was no correlation between the percentage of demonstrated STF as measured by the two presentation methods (*r*(39) = 0.01, *p* = 0.96). However, there was a significant correlation between the percentage of demonstrated TTFS as measured by the two methods (*r*(39) = 0.39, *p = 0*.02).

To further compare computer and object presentations, children were divided into two groups based on their performance in the computer presentation: children who recorded at least one STF (*n* = 26) and children who recorded no instances of STF—(*n* = 13, six failed to calibrate and seven calibrated but recorded no STF).

When results were cross-tabulated with their performance on the STF object presentation task (Table 3), it was noted that all 13 children who recorded no instances of STF on the computer presentation demonstrated STF on the object presentation tasks. These children demonstrated STF on an average of 85% of trials (*SD* = 18.7%, *Range* = 40–100%). Six of these children (46%) demonstrated STF on all 10 trials in the object presentation task.

The same comparison was performed for the TTFS task, with children divided into groups based on their performance in the computer presentation: those who recorded at least one TTFS (*n* = 18) and those who recorded no instances of TTFS (*n* = 21, six failed to calibrate and 15 calibrated but recorded no TTFS).

When results were cross-tabulated with their performance on the object presentation of the TTFS task (Table 4), it emerged that 16 children who showed no instances of TTFS on the computer-presented task could show at least one TTFS on the object-based presentation. These children demonstrated TTFS on an average of 80% of object-based trials (*SD* = 24%, *Range* = 25–100%).

## 4. Discussion

This study explored two different methods of observing and recording single-target fixation (STF) and target–target fixation shift (TTFS) in children with CP: presenting stimuli on a computer display connected to an eye tracker, and presenting objects with observations of gaze made by two independent observers. Whilst the two presentations are not identical, the results of this comparative study offer some insight into the utility of both methods to clinicians observing children’s use of directed gaze.

In the case of both STF and TTFS, the object presentation of the task resulted in significantly more instances of the target gaze behaviours than the computer presentation. It is not possible from the data collected in this study to answer definitively the question of which method delivers the “correct” result; however the difference between the scores on the two presentations is notable and worthy of discussion—with the scores on the object-based task being significantly higher. In the case of the computer presentation, the difficulties with calibration experienced by some children and the accuracy requirements for the eye tracker to determine a fixation might have resulted in the device under-reporting the number of fixations that would have been recorded by human observers. It is also possible that the observers in the object presentation may be over-reporting instances of the target gaze behaviours, either through being primed to look for fixations or gaze shifts, or perhaps accepting a higher “margin of error” than might an eye tracker. It is striking that a number of children demonstrated multiple instances of both STF and TTFS on the object presentation task, but recorded no instances of either on the computer presentation task. This is interesting, given that the duration required to constitute a fixation was set longer on the object presentation (2 s) than on the computer presentation (1 s). The aim of setting a longer duration was to increase the confidence of the observers that a defined fixation had taken place: they would only report a fixation if they were sure and would be less likely to report a fleeting glance towards the target as a fixation. If anything, this longer duration requirement might be expected to result in fewer reports of fixation compared with the shorter duration requirement of the computer-presented task. Instead we found the opposite. It is, of course, possible that the human observers did not stick to this rule—it is not possible to assess this from our data—however, the level of agreement between the two independent observers makes this less likely. Object presentations were scored independently by two observers, who achieved moderate to substantial agreement. If these observers were over-reporting the observed gaze behaviours, this agreement would indicate that they were doing so in parallel, scoring the same types of observed behaviours as fixations or gaze shifts, or that they were applying similar interpretations to what they saw. This would, in turn, suggest that there is at least something specific about these behaviours. Since both observers had clinical experience in the observation and testing of fixations, one interpretation might be that they were more willing to accept a less precise fixation or shift, as long as it fulfilled their own concept of the functional requirements of this behaviour. One possible area of future enquiry would be to have observers resolve any disagreements subsequent to the object-based tasks, perhaps using a video recording. However, there are complications in comparing live and post-hoc recorded instances, and the assessment of gaze direction and fixation from video recordings can be challenging [5].

Another possibility is that the object presentation is a more “social” task, and this is what motivates gaze behaviours. Although every effort was made to conceal the assessor, who manipulated the objects from behind a screen, children are likely to have been aware of a person’s presence and that this person was moving the objects. Children may have engaged more with this presentation as a result of perceiving it to be more social or more communicative than the computer-based equivalent. However, social and communicative contexts cannot be assumed to be a motivator for all children, particularly not all children with CP. For example, a recent study by Price and colleagues [59] showed that around 30% of a sample of non-speaking children with bilateral CP (*n* = 32) showed limited engagement in routines designed to elicit social communication (joint attention and social orienting) as measured by observations of their gaze behaviours; however, the same children were able to demonstrate relatively intact fixation and gaze shifting skills on a non-social task assessed using the same procedure as the object presentation used in this study. Such a finding underlines the importance of assessing fixation skills in a non-social context: doing so can help rule out the possibility that a general absence of directed gaze behaviours can be explained by the child’s profile of early social communication skills.

The presentation of objects, in general, has the advantage of being more flexible in a clinical setting. Whilst the protocol in this study was standardised as best as possible, so that objects were presented for a defined amount of time and in specific locations, the use of objects in assessment and structured observations presents clinicians with opportunities to present children with stimuli in varying locations, at varying distances and for different durations [30]. This is a particular advantage in the case of children with more severe CP, who frequently present with difficulties with head control or controlling movement and posture.

Regarding the computer presentation of the task, the research question of whether children enrolled in the study were able to calibrate an eye tracker with sufficient precision to allow reliable collection of gaze data produced some interesting results and highlighted some variable performance within the group. The calibration rate of 84.6% is lower than rates reported in the literature for typically developing children (92–100%) [39] and similar to those reported for children with intellectual disability (83%) and Downs syndrome (88%) [39,45]. Whilst these are not matched groups, it is interesting to observe the similar calibration rates between participants in this study and children without significant movement limitations in other studies. It is also interesting to note that successful calibration was not related either to language age or degree of movement limitation. Using an eye tracker to gather gaze point data requires calibration to be successful in order for the data to be valid. In this sense, calibration acts as an “entry barrier” to completing the directed gaze tasks, meaning that some children whose fixations might have benefited from the investigation would be excluded by the need for accuracy imposed by the technology. Taking the view of a clinician seeking to assess these skills in children with CP, it may be tempting to say that failure to calibrate reflects a general inability to fixate on a single target. Nyström and colleagues [46] propose that, whilst calibration is a single-object fixation task, it is one that has extremely high accuracy requirements, as the purpose of calibration in eye tracking is to ensure the validity of collected data. The accuracy threshold for calibration may simply be too high for some of the children in this group, which may explain why all six children who were unable to calibrate the eye tracker could nevertheless demonstrate instances of fixation on the object presentation task.

The questions of accuracy in the computer presentation and interpretation in the object presentation may offer a possible future area for investigation. One potential limitation of the current study is the use of areas of interest in the analysis phase that were sized to the same area as the stimuli and the use of the default fixation threshold. Both of these were justified methodologically as offering the most objective recording possible of these gaze behaviours. Given the results of the current study; however, there is the possibility to explore changing some of these parameters, to allow the setting of different criteria for fixation duration or accuracy, which could mirror the margin of error considered acceptable by human observers. Similarly, the use of an alternative fixation algorithm may be an area for further exploration; however, the default fixation algorithm was used in this study to provide a point of comparison. Lowering the calibration requirements may reduce or remove the entrance barrier for these tasks for some children with CP. Additionally, although a lower calibration threshold may reduce our confidence in the accuracy of the gaze point data, a comparison with the object-based presentation suggests that this might be an acceptable trade-off for those needing a way to observe gaze behaviours. Other calibration paradigms [60,61] may prove more flexible, and this is an area where future research is needed for the population of children with CP and other physical disabilities [62]. There is also the possibility of expanding this study beyond the recording of data into the field of eye-gaze control. An eye-gaze control device has a lower calibration and accuracy threshold than an eye tracker and could also provide feedback to the user on whether fixation has been achieved [63,64].

One area where this could be particularly useful is the group of children presenting with strabismus. The results of this study show that children with strabismus (*n* = 12) performed similarly to those without strabismus on the computer presentation of both the STF and TTFS tasks. The mean performance of this group on the object-based presentation was lower than the mean of the group without strabismus for both tasks. Although the difference was not significant, the large effect size may suggest that observers are more influenced by the presence of strabismus. The results of this study are consistent with the published literature, which suggests that clinicians should ensure they understand the nature of a child’s eye movements when observing fixations, ensuring that they are confident in establishing the dominant eye and offering extra time to establish fixations [65]. Eye trackers, with their high spatial resolution and ability to provide gaze point data for each eye independently, can be used to screen, recognise and quantify strabismus [66,67]. Eye-gaze control systems can be adapted to account for strabismus, either by tracking only the dominant eye or by factoring in the difference in gaze point at the calibration stage. Using eye-gaze control technology to support the observation of fixation in this group of children is a possible area for future study.

There are further clinical implications to these findings in that they highlight the need for clinicians to be cautious of the de facto assumption that computer presentations of stimuli are “better” or more accurate in identifying and recording children’s fixations. Whilst it is too early to say whether computer-based or object-based methods should be preferred, the results of this study show that object presentation scored reliably and that the computer presentation may, in fact, underestimate fixations in some children with CP. Further exploration and comparison of these methods is indicated as an area of future research.

Irrespective of the method used to elicit fixations, what emerges is a picture of a population of children whose use of functional vision and targeted fixation is variable. This variability does not seem to be wholly explained by children’s language age equivalent, the severity of their gross motor impairment as measured using GMFCS, or the presence or absence of strabismus. In this sense, the results of the present study contribute to the growing body of work [20,30,59,63], which underlines the importance of assessing these skills in children who may be expected to use their gaze either for signalling messages or for control of a computer or other assistive technology. The variability observed within the group highlights that the presence and functional use of gaze behaviours should not be assumed to be present in any particular group of children and adds more weight to the idea that these skills are important to measure and understand when working with individual children.

## 5. Conclusions

This paper offers insight into the question of whether computer or object presentation of stimuli is better suited to the task of providing clinicians with information about fixation and gaze shifting in children with CP. The results do not conclusively endorse either method in isolation but highlight that each may have strengths and weaknesses. Eye tracking provides a highly accurate method of recording these gaze behaviours. There is a tendency to assume that the high levels of accuracy offered by an eye tracker will result in a more “objective” method of measuring or recording certain skills. However, the data from the current study suggest that using an eye tracker in isolation presents a risk of missing skills that children are able to demonstrate by other means. Conversely, the observation of children’s gaze towards objects, as scored by human observers, may be less accurate but may also be a closer approximation of what clinicians consider to be functional fixations in this population of children.

## Figures and Tables

**Table 1:** Participant Characteristics.

	Participants	
	*n*	% of Category
Total	39	100.0
Gender		
Male	21	53.8
Female	18	47.2
CP Type		
Dyskinetic	20	51.3
Spastic	2	5.1
Mixed	8	20.5
Unspecified	9	23.1
Reported Visual Issues		
Strabismus	12	30.1
Dysmorphologies (e.g., astigmatism)	1	2.6
GMFCS Level ^1^		
IV	15	38.5
V	24	61.5

^1^ Gross Motor Function Classification Scale [53].

**Table 2:** Chronological, language and non-verbal ages of participants (months).

	Mean	SD	Range
Chronological Age	91.11	30.35	40–145
Language Age Equivalent	26.24	12.30	11–54
Non-Verbal Cognitive Age Equivalent	25.57	13.06	10–54

**Table 3:** Comparison of computer and object presentations on STF task.

	No STF on Object Presentation	≥1 STF on Object Presentation	Total
No STF on computer presentation	0	13	13
≥1 STF on computer presentation	2	24	26
Total	2	37	39

**Table 4:** Comparison of computer and object presentations on TTFS task.

	No TTFS on Object Presentation	≥1 TTFS on Object Presentation	Total
No TTFS on computer presentation	5	16	21
≥1 TTFS on computer presentation	0	18	18
Total	5	34	39

## Data Availability

The raw data supporting the conclusions of this article will be made available by the authors on request.

## Abbreviations

The following abbreviations are used in this manuscript:

AAC	Augmentative and alternative communication
AOI	Area of interest
CP	Cerebral palsy
GMFCS	Gross Motor Function Classification Scale
MDPI	Multidisciplinary Digital Publishing Institute
MSEL	Mullen Scales of Early Learning
PLS-4UK	Pre-School Language Scales UK—4th Edition
RTF	Reaction Time to Fixation
STF	Single-target fixation
TTFS	Target–target fixation shift
